# A systematic review and meta-analysis of acupuncture for improving learning and memory ability in animals

**DOI:** 10.1186/s12906-016-1298-3

**Published:** 2016-08-19

**Authors:** Kai-Yu Huang, Shuang Liang, Mei-Ling Yu, Shu-Ping Fu, Xia Chen, Sheng-Feng Lu

**Affiliations:** 1 The No.2 Clinical Medical College, Nanjing University of Chinese Medicine, Nanjing, 210023 China; 2 Key Laboratory of Acupuncture and Medicine Research of Ministry of Education, Nanjing University of Chinese Medicine, Nanjing, 210023 China

**Keywords:** Acupuncture, Learning, Memory, Meta-analysis

## Abstract

**Background:**

Memory loss is the most prominent symptoms of brain aging, but there is currently no evidence-based treatment strategy. Acupuncture has been widely used in China and the effectiveness for improving learning and memory has been mentioned in previous studies. We conducted this systematic review and meta-analysis to evaluate the effectiveness of acupuncture for improving learning and memory in animal experiments.

**Methods:**

We searched Pubmed, Embase, Ovid Medline(R), the China National Knowledge Infrastructure (CNKI), Chinese Science and Technology Periodical Database (VIP) and Wanfang data Information Site to collect studies published up to December 2015. Study quality for each included article was evaluated according to the CAMARADES 10-item checklist. Outcome measure is Morris water maze. A meta-analysis was conducted according to the Cochrane systematic review method by using RevMan 5.3 software.

**Results:**

Forty-two studies involving 944 animals were included. The quality score of the studies ranged from 2 to 8, with a mean of 5.3. Meta-analysis results showed that 24 studies reported significant effect of acupuncture for decreasing escape latency (−3.00, 95 % CI: −3.78 ~ −2.23, *P* < 0.00001), 14 studies reported significant effect of acupuncture for increasing frequency of cross platform (2.57, 95 % CI: 1.92 ~ 3.22, *P* < 0.00001), and 7 studies reported significant effect of acupuncture for increasing time in target quadrant (2.00, 95 % CI: 1.10 ~ 2.91, *P* < 0.00001) compared with the control group.

**Conclusions:**

These findings show acupuncture has a potential role in improving learning and memory ability in animal models, suggesting it as a candidate therapy for memory loss of aged brain.

## Background

Learning and memory are the most basal and important higher nervous functions and closely related to each other [1]. Learning means accepting information from the outside environment and memory refers being able to use this information at a later date [2–4]. As a symptom, learning and memory impairment often appears together in some diseases, such as Alzheimer’s disease, vascular dementia, diabetes, autism, and so on [5–8]. With the increasing of living pressure and changing in life style, learning and memory impairment as the important embodiment of brain dysfunction have become one of the most important factors that affect people’s lives [1, 2]. The treatment based on western medical science for most learning and memory impairment only relieves the symptoms and delays the progression of disease [9, 10]. Moreover, it also has some side effects caused by long time treatment [11].

As a kind of economical and side-effect free natural remedies, acupuncture has been used in China widely for over 2000 years [12]. The effect of acupuncture on encephalopathy has been recognized internationally. More and more studies have been published to confirm the effectiveness of acupuncture for improving learning and memory [13–16]. However, to some extent, the small sample size makes it hard to draw firm conclusions.

Up to now, there have been no systematic reviews to analyze the effectiveness of acupuncture for improving learning and memory. Reviews based on animal data could make trails’ planning more perfect, increase the odds of success of future clinical trials and assist to decide what is valuable in further research [17]. Additionally, animal experiment can make us better understand the mechanism of acupuncture on learning and memory and guide the future clinical study. Therefore, we conducted a systematic review and meta-analysis of the effectiveness of acupuncture for improving learning and memory in animal experiments to provide suggestions for future animal experiments and clinical trials.

## Methods

### Search strategy

The following electronic databases were searched: Pubmed, Embase, Ovid Medline(R), China National Knowledge Infrastructure (CNKI), Chinese Science and Technology Periodical Database (VIP) and Wanfang data Information Site. The publication time is from the inception of each database up to December 2015. The languages were limited to English and Chinese. Search terms consisted of two groups: intervention (acupuncture and other related terms) and object (learning and memory and other related terms). All searches were limited to animals. We combined the results of all searches and then removed the duplicates. We also tried to get additional records identified through other sources.

### Inclusion criteria

They were included if the following criteria were met:

1. Subjects:Animal models of learning and memory impairment were included.
2. Interventions: Acupuncture was the main therapy and only included manual acupuncture and electroacupuncture.
3. Outcomes: Morris water maze test was the primary outcome to explore the effectiveness of acupuncture groups and the difference between control groups and acupuncture groups. The Morris maze test is arguably the preferred test for assessing learning and memory in basic research. As a classic test, it has been accepted and used widely in most related animals experiments.
4. Language: Chinese and English articles.

### Exclusion criteria

They were excluded if the following criteria were met: (1) Scalp acupuncture, auricular acupuncture, moxibustion and other forms of acupuncture; (2) studies that included Chinese herbal medicine or Western medicine; (3) studies that compared different acupuncture techniques or different acupoints; (4) studies without control group; (5) duplicate publications.

### Study selection and data extraction

According to the above design, one reviewer (KYH) searched those databases and listed the titles of all articles. Two evaluators (KYH and SL) assessed the eligibility of these articles and made decision on every research (inclusion or exclusion) independently. If they did not reach the same decision, the concerned articles were discussed with a third reviewer (SFL).

Two reviewers (KYH and SL) extracted data independently from each study. The database included: (1) basic information, including publication year, the first author’s name and model of learning and memory impairment; (2) individual data, including the number of animals, species and weight in acupuncture group and control group; (3) information on treatment, including timing and duration for treatment and method of treatment procedure; (4) the results of Morris water maze test. If outcomes were presented at different time points, we extracted data from the last time point. Differences of extracted data were solved after discussion with a third reviewer (SFL).

### Quality assessment

We evaluated the methodological quality of the included studies by a ten-item scale [12]: (1) publication in a peer-reviewed journal; (2) statements describing control of temperature; (3) random allocation to treatment or control; (4) blinded building of model; (5) assessment whether building model is successful; (6) blinded assessment of outcome;(7) use of anesthetic without significant intrinsic neuroprotective activity; (8) sample size calculation; (9) compliance with animal welfare regulations; (10) declared any potential conflict of interest. Each item of the ten-item scale was attributed to one point. Based on this, each study had a quality score from zero to ten. The higher the score is, the better the article’s quality is.

Two reviewers (KYH and SL) extracted data independently and assessed study quality. Disagreements were solved after discussion with a third reviewer (SFL).

### Statistical analysis

Some results of Morris water maze test including escape latency, frequency of cross platform and time in target quadrant were considered as continuous data. Standard mean difference (SMD) was given, which was an estimate of the combined effect sizes. Publication bias was assessed with a funnel plot. Moreover, to explore the impact of factors affecting the outcome measures, we analyzed the specific subgroups based on escape latency and frequency of cross platform for the following factors: manual acupuncture and electroacupuncture, articles published or unpublished, species of animals, different ways to make Alzheimer’s disease (AD) model and different ways to make vascular dementia (VD) model.

The meta-analysis was performed with RevMan 5.3 software. The confidence interval (CI) was established at 95 %, and P values of less than 0.05 were considered statistically significant. For the assessment of heterogeneity, the I^2^ statistic and ***χ***^2^ distribution were used.

## Results

### Study inclusion

**Fig. 1:**
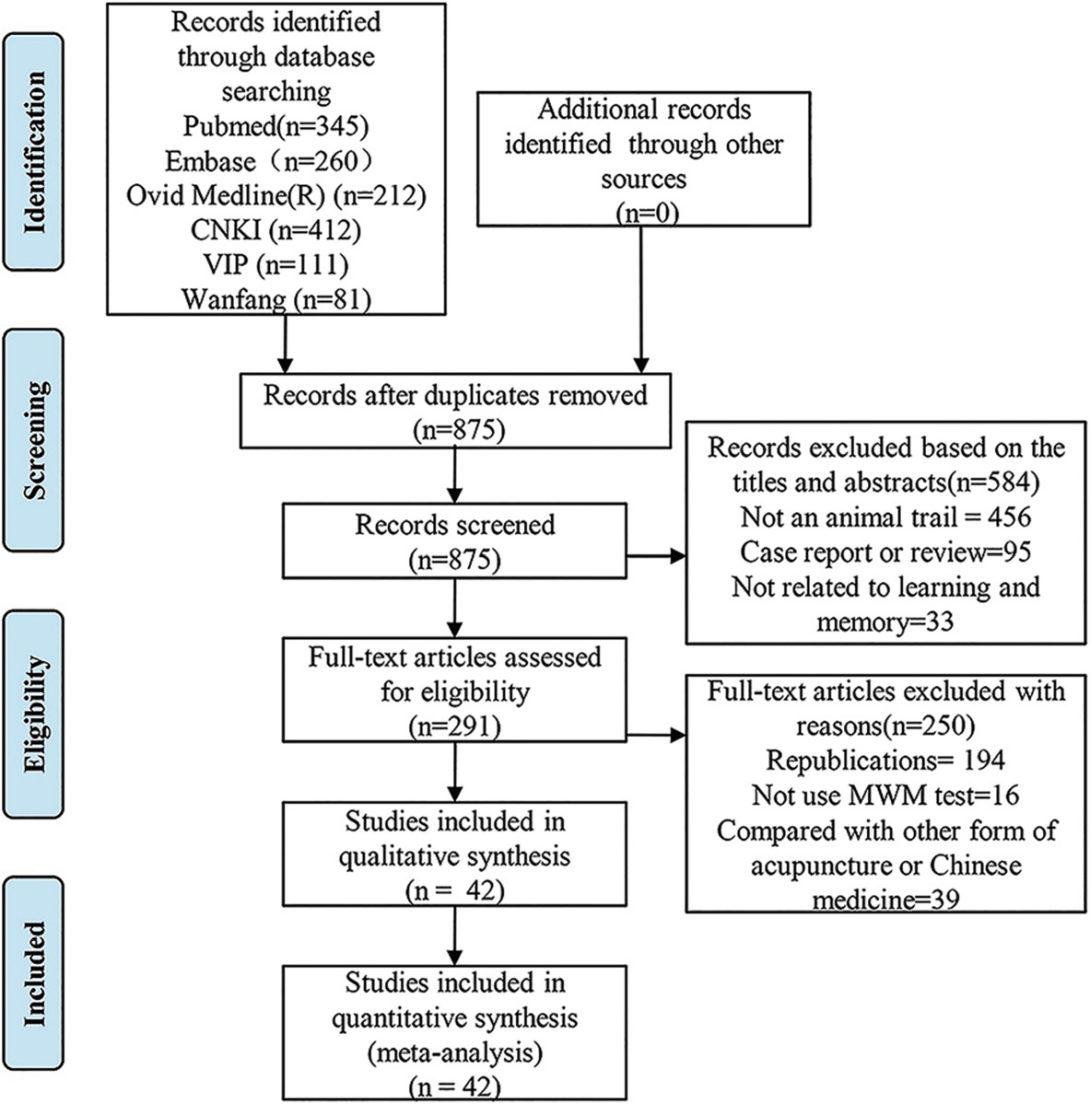
Flow diagram of the study selection process for this systematic review and meta-analysis

Initially, 1421 records were searched from six databases. After removing duplicates, the records were decreased to 875. Based on titles and abstracts of records, we excluded 584 papers with reasons, such as not an animal experiment, case report or review, not related to learning and memory, and so on. The 291 remaining articles were downloaded for further selection. Due to republications, not using the Morris water maze test, comparing with other forms of acupuncture or Chinese herbs, and so on, 250 articles were excluded. Eventually, 42 studies were included [18–59]. The flow diagram of the study selection process is shown in Fig. 1.

### Study characteristics

The 42 included studies involved 944 rats. The total animal number in control groups is 470 and the number in acupuncture groups is 474. 36 studies of all have mentioned specific weight of rats. The rats’ weight ranged from 160 to 320 g in 29 studies. The rats’ weight was around 20 g in 3studies and more than 320 g in 4 studies. The age of animals was different and mentioned concretely in 20 studies. It ranged from new-born to 24-month-old. 1 study used new-born rats; 10 studies used 2–4 months old rats; 6 studies used 6–9 months old rats; 3 studies used aged rats (more than 12 months old). Different subtests of the Morris water maze test were used in these studies: 41 studies with 912 animals reported data as escape latency, 18 studies with 406 animals reported data as frequency of cross platform and 12 studies using 255 animals reported data as time in target quadrant. The rat species included Sprague–Dawley (SD) rats, Wistar rats and AKR rats. Eighteen out of the 42 studies (42.9 %, *n* = 377) were AD models. Fourteen studies (33.3 %, *n* = 309) were VD models. And the 10 remaining studies (23.8 %) used other models. The main characteristics of the 42 studies are shown in Table 1.

**Table 1:** Characteristics of the included studies

Study	Species (Nc/Na)	Weight(g)	Age (month)	Model	Acupuncture (acupoints)	Control intervention	Outcome index	*P* value
Bao 2014 [18]	SD Rats (12/12)	200 ± 20	NR	PTSD (CMS)	Electroacupuncture 20 min/d for 21d, continuous waves of 2Hz of frequency and current density of 2 mA (GV20, GV29).	Fluoxetine	1. escape latency 2. total swimming distance	1. *P* < 0.01 2. *P* < 0.01
Zeng 2008 [19]	Wistar Rats (10/10)	250 ± 10	NR	AD (D-gal, NaNO_2_)	Electroacupuncture 20 min/d for 60d, disperse- dense waves of 80/100 Hz of frequency (LI4, LR3).	Donepezil	1. escape latency	1. *P* < 0.01
Chen 2015 [20]	SD Rats (13/14)	230 ± 20	NR	VD (4-VO)	Electroacupuncture 30 min/d for 7d, disperse- dense waves of 1/20 Hz of frequency (GV24, GV20).	NR	1. escape latency 2.total swimming distance 3. frequency of cross platform	1. *P* < 0.05 2. *P* < 0.05 3. *P* < 0.05
Chen 2006 [21]	Wistar Rats (34/34)	NR	New- born	HIBD (closed space)	Manual acupuncture 20 min/d for 10d (GV20, GV14).	NR	1. escape latency 2. percentage of time in target quadrant 3. percentage of swimming distance in target quadrant	1.*P* < 0.05 2. *P* < 0.05 3. *P* < 0.05
Dai 2015 [22]	AKR Rats (10/10)	24.0 ± 3.5	6	AD (SAMP8)	Manual acupuncture 10 min/d for 28d (GV20, SP10, BL17, BL23).	NR	1. escape latency 2. frequency of cross platform 3. time in target quadrant	1. *P* < 0.05 2. *P* < 0.05 3. *P* < 0.05
Hou 2013 [23]	SD Rats (10/10)	220 ± 20	NR	PTSD(SPS)	Electroacupuncture 30 min/d for 7d, continuous waves of 2Hz of frequency and current density of 1 mA (GV20, ST36).	NR	1. escape latency	1. *P* < 0.05
Gao 2012 [24]	SD Rats (16/16)	400–500	20–24	AD(aged rats)	Electroacupuncture 30 min/d for 21d, disperse- dense waves of 2/100 Hz of frequency and intensity of 2–4 V (GV20, KI1).	NR	1. swimming time	1. *P* < 0.05
Huang 2010 [25]	Wistar Rats (10/10)	200 ± 20	NR	PD (6-OH DA)	Electroacupuncture 30 min/d for 24d, continuous waves of 100Hz of frequency and current density of 0.5 mA (GV16,LR3).	Madopar	1. escape latency	1. *P* < 0.01
Ji 2011 [26]	SD Rats (10/10)	220–260	NR	VD (4-VO)	Manual acupuncture 30 min/d for 30d (GV20, CV17, CV6, BL17, SP6).	NR	1. escape latency 2. frequency of cross platform	1. *P* < 0.01 2. *P* < 0.01
Jia 2011 [27]	SD Rats (20/20)	220 ± 20	NR	VD (2-VO)	Electroacupuncture 20 min/d for 14d, continuous waves of 2Hz of frequency and intensity of 3 V (GV20, GV14).	NR	1. escape latency 2. frequency of cross platform	1. *P* < 0.05 2. *P* < 0.05
Wang 2012 [28]	Wistar Rats (10/10)	300 ± 10	NR	AD(STZ)	Electroacupuncture for 28d (GV20, GV14, ST36).	NR	1. escape latency 2. frequency of cross platform 3. time in target quadrant	1. *P* < 0.05 2. *P* < 0.05 3. *P* < 0.05
Lin 2008 [29]	SD Rats (10/10)	620 ± 80	12	VD (2-VO)	Electroacupuncture 20 min/d for 30d, continuous waves of 2Hz of frequency and current density of 1–2 mA (GV20, GV14,BL23).	Sham acupuncture	1. escape latency 2. time in target quadrant	1. *P* < 0.05 2. *P* < 0.05
Luo 2007 [30]	SD Rats (14/14)	200 ± 20	NR	VD (4-VO)	Electroacupuncture 20 min/d for 15d, continuous waves of 150Hz of frequency and current density of 1 mA (GV20, BL17, BL20, BL23).	Nimodipine	1. escape latency	1. *P* < 0.01
Ma 2009 [31]	Wistar Rats (13/13)	200–250	NR	Diabete (STZ)	Electroacupuncture 15 min/d for 14d, continuous waves of 0.5Hz of frequency and current density of 30 mA (GV20, GV14).	NR	1. escape latency	1. *P* < 0.05
Zhang 2014 [32]	AKR Rats (10/10)	NR	4	AD (SAMP8)	Manual acupuncture 30 min/d for 30d, twisting 10 s a time (GV20,ST36).	NR	1. escape latency 2. frequency of cross platform 3. time in target quadrant	1. *P* < 0.05 2. *P* < 0.05 3. *P* < 0.05
Niu 2009 [33]	SD Rats (10/10)	300 ± 20	NR	VD (4-VO)	Electroacupuncture 10 min/d for 42d, disperse- dense waves of 80/100 Hz of frequency and current density of 1–3 mA (GV29, LI20).	NR	1. escape latency 2. frequency of cross platform	1. *P* < 0.01 2. *P* < 0.01
Su 2013 [34]	SD Rats (12/12)	200–250	NR	AD(D-gal, Aβ1- 42)	Electroacupuncture 15 min/d for 28d, continuous waves of 35Hz of frequency and intensity of 2 V (GV20, KI3, ST36).	NR	1. escape latency 2. frequency of cross platform 3. time in target quadrant	1. *P* < 0.05 2. *P* < 0.05 3. *P* < 0.05
Tan 2014 [35]	Wistar Rats (8/8)	250 ± 50	2	VD (MCAO)	Electroacupuncture 15 min/d for 21d, continuous waves of 16Hz of frequency and current density of 1 mA (GV20, GV14).	NR	1. escape latency 2. frequency of cross platform 3. percentage of time in target quadrant	1. *P* < 0.05 2. *P* < 0.05 3. *P* < 0.05
Tang 2014 [36]	SD Rats (10/10)	160–200	3	OVX	Electroacupuncture 20 min/d for 45d, continuous waves of 3–4Hz of frequency and current density of 4–5 mA (ST36, BL23).	Sham acupuncture	1. escape latency 2. frequency of cross platform	1. *P* < 0.01 2. *P* < 0.01
Wang 2013 [37]	SD Rats (10/10)	200 ± 20	NR	AD (D-gal, NaNO_2_)	Manual acupuncture 10 min/d for 30d (LI4, LR3, ST36).	Donepezil	1. escape latency 2. frequency of cross platform	1. *P* < 0.01 2. *P* < 0.01
Wang 2009 [38]	SD Rats (12/13)	240 ± 20	3	VD (2-VO)	Manual acupuncture 10 min/d for 30d (GV20, BL17, CV6, SP6, CV17).	Piracetam	1. escape latency 2. frequency of cross platform	1. *P* < 0.01 2. *P* < 0.05
Hong 2014 [39]	Wistar Rats (10/10)	300–350	NR	Autism (VPA)	Manual acupuncture 1 min/d for 30d (GV1).	Sham acupuncture	1. escape latency 2. average speed	1. *P* < 0.05 2. *P* < 0.05
Xu 2006 [40]	SD Rats (13/14)	200–220	2	VD (4-VO)	Electroacupuncture 20 min/d for 20d, continuous waves of 150Hz of frequency and current density of 20 mA (GV20, GV14).	Nimodipine	1. escape latency 2. frequency of cross platform	1. *P* < 0.01 2. *P* < 0.01
Xu 2007 [41]	SD Rats (8/10)	180–200	NR	AD (D-gal)	Electroacupuncture 20 min/d for 21d, continuous waves of 3Hz of frequency and current density of 1 mA (GV20, ST36).	NR	1. escape latency 2. percentage of swimming distance in target quadrant 3. percentage of time in target quadrant	1. *P* < 0.01 2. *P* < 0.01 3. *P* < 0.01
Yi 2014 [42]	SD Rats (12/12)	200 ± 34	4	AD (Aβ25–35)	Electroacupuncture 30 min/d for 12d, disperse- dense waves of 2/30 Hz of frequency and current density of 1 mA (GV29, LI20).	NR	1. escape latency 2. frequency of cross platform 3. time in target quadrant	1. *P* < 0.05 2. *P* < 0.05 3. *P* < 0.05
Yu 2014 [43]	Wistar Rats (10/10)	200–250	NR	WD (CuSO4)	Electroacupuncture 15 min/d for 7d, continuous waves of 2Hz of frequency and current density of 1 mA (HT7).	NR	1. escape latency	1. *P* < 0.01
Feng 2013 [44]	AKR Rats (10/9)	29–35	9	AD (SAMP8)	Manual acupuncture 20 min/d for 28d, twisting 2.5 times/s for 60 s(GV20, KI1).	NR	1. escape latency 2. frequency of cross platform	1. *P* < 0.05 2. *P* < 0.05
Li 2013 [45]	Wistar Rats (10/10)	200–250	4	AD(STZ)	Electroacupuncture for 28d, continuous waves of 30Hz of frequency and intensity of 2 V (BL23, KI3, ST36, GV20, GV14).	Donepezil	1. escape latency 2. frequency of cross platform	1. *P* < 0.05 2. *P* < 0.05
Wang 2013 [46]	AKR Rats (10/10)	NR	8	AD (SAMP8)	Manual acupuncture for 15d (CV17, CV12, CV6, SP10,ST36).	Sham acupuncture	1. escape latency 2. time in target quadrant	1. *P* < 0.01 2. *P* < 0.01
Zheng 2009 [47]	Wistar Rats (8/7)	212 ± 15	2	VD (2-VO)	Electroacupuncture for 28d, continuous waves of 2Hz of frequency (GV20, KI3).	NR	1. escape latency	1. *P* < 0.05
Li 2012 [48]	AKR Rats (15/15)	NR	7.5	AD (SAMP8)	Manual acupuncture for 15d, twisting 2 times/s for 30 s (CV17, CV12, CV6, SP10, ST36).	Sham acupuncture	1. escape latency 2. time in target quadrant	1. *P* < 0.05 2. *P* < 0.05
Li 2014 [49]	C57BL/6 Rats (6/6)	NR	2	AD (APP/PS1)	Electroacupuncture 30 min/d for 20d, disperse- dense waves of 2/15 Hz of frequency and current density of 1 mA (GV20).	NR	1. escape latency	1. *P* < 0.05
Lee 2014 [50]	SD Rats (7/7)	220–240	NR	AD(SCO)	Manual acupuncture for 15d (GV20).	Sham acupuncture	1. escape latency 2. swimming speed 3. percentages of time in a probe trial	1. *P* < 0.05 2. *P* < 0.05 3. *P* < 0.05
Zhu 2013 [51]	SD Rats (6/6)	432 ± 30	12	VD (2-VO)	Electroacupuncture 20 min/d for 30d, continuous waves of 4Hz of frequency and current density of 2 mA (GV20, GV14, BL23).	NR	1. escape latency	1. *P* < 0.05
Lu 2014 [52]	SD Rats (8/8)	200–250	NR	Ethanol	Electroacupuncture 20 min/d for 30d, continuous waves of 2Hz of frequency and current density of 1.5–2 mA (ST36).	Sham acupuncture	1. escape latency 2. time in target quadrant	1. *P* < 0.05 2. *P* < 0.05
Li 2012 [53]	SD Rats (10/10)	250 ± 30	3	VD (MCAO)	Electroacupuncture 30 min/d for 14d, disperse- dense waves of 2/30 Hz of frequency and current density of 2 mA (GV20, GV14).	NR	1. escape latency	1. *P* < 0.01
Guo 2015 [54]	SD Rats (10/10)	250–300	NR	AD (Aβ1–40)	Electroacupuncture 30 min/d for 24d, continuous waves of 20Hz of frequency and current density of less than 2 mA (GV20, BL23).	Sham acupuncture	1. escape latency 2. time in target quadrant 3. frequency of cross platform	1. *P* < 0.01 2. *P* < 0.01 3. *P* < 0.01
Jiang 2015 [55]	AKR Rats (10/10)	NR	7.5	AD (SAMP8)	Electroacupuncture for 14d, continuous waves of 2Hz of frequency,current density of 0.6 mA and intensity of 2 V (GV20,GV26, GV29).	NR	1. escape latency 2. percentages of time in target quadrant	1. *P* < 0.01 2. *P* < 0.01
Shao 2008 [56]	SD Rats (8/9)	180–220	NR	VD (4-VO)	Electroacupuncture 20 min/d for 15d, continuous waves of 150Hz of frequency and current density of 1–2 mA (GV20, BL17, BL20, BL23).	Nimodipine	1. escape latency 2. time in target quadrant	1. *P* < 0.01 2. *P* < 0.01
Liu 2013 [57]	SD Rats (12/12)	200 ± 20	NR	CFS	Manual acupuncture 20 min/d for 21d, twirling reinforcing (ST36).	NR	1. escape latency 2. frequency of cross platform	1. *P* < 0.05 2. *P* < 0.05
Li 2015 [58]	Wistar Rats (11/11)	320–360	NR	VD(micro- emboli)	Manual acupuncture for 12d, twisting 2 times/s for 30 s (ST36).	Sham acupuncture	1. escape latency	1. *P* < 0.01
Lu 2008 [59]	AKR Rats (12/12)	20 ± 2	8	AD (SAMP8)	Electroacupuncture 20 min/d for 7d, disperse- dense waves of 2/100 Hz of frequency and intensity of 2–4 V (GV20, KI1).	NR	1. escape latency 2. time in target quadrant	1. *P* < 0.05 2. *P* < 0.05

*Nc* animal number in control group, *Na* animal number in acupuncture group, *PTSD* post-traumatic stress disorder, *CMS* chronic mild stimulation, *NR* not report, *AD* Alzheimer’s disease, *VD* vascular dementia, *4*-*VO* 4- vessel occlusion, *SPS* single prolonged stress, *PD* Parkinson’s disease, *6*-*OHDA* 6-OH-dopamine, *STZ* streptozotocin, *CFS* chronic fatigue syndrome, *MCAO* middle cerebral artery occlusion, *OVX* ovariectomy, *VPA* sodium valproate, *WD* Wilson disease

### Description of acupuncture regime

Varied acupuncture techniques were used in terms of selection of acupuncture-points, manipulation or stimulation methods (Table 1). The most commonly used acupoints, which have been used by four or more studies, were GV20 (baihui), ST36 (zusanli), GV14 (dazhui), BL23 (shenshu), BL17 (geshu) and CV17 (danzhong). The frequency of acupuncture was mostly once per day. Animals received acupuncture treatment 1 to 30 min per session. The course of acupuncture treatment ranged from 7 to 60 days. The average duration of acupuncture was 22.4 days (SD = 10.8). 13 studies used manual acupuncture, and the rest 29 studies chose electroacupuncture. 5 of 13 studies stated detailed operating methods of manual acupuncture. The operating method of manual acupuncture was mostly twirling reinforcing. 28 of 29 studies stated detailed operating parameters of electroacupuncture and only one ignored related descriptions. 20 of 28 studies used continuous waves. The frequency of continuous wave is from 0.5Hz to 150Hz. The current density of continuous wave is from 0.5 mA to 30 mA. The rest 8 studies used disperse-dense waves. The frequency of disperse wave is from 1Hz to 80Hz and the frequency of dense wave is from 15Hz to 100Hz. The current density of continuous wave is from 1 mA to 3 mA.

### Description of control interventions

Eighteen of included studies used some interventions in control groups (Table 1). Control interventions consisted of western medicine and sham acupuncture. Western medicine was adopted in 9 studies and 9 experiments used sham acupuncture. Medication was administered for similar treatment duration as acupuncture. Types of control medication consisted of donepezil (3 studies), nimodipine (3 studies), fluoxetine (1 study), madopar (1 study) and piracetam (1 study). Puncturing points lateral to acupoints is the way of sham acupuncture in 8 experiments. Not using electroacupuncture apparatus is the other way of sham acupuncture in 1 experiment which adopted electroacupuncture as the intervention way.

### Study quality and publication bias

The score of the study quality was ranged from 2 to 8 out of a total 10 points. Concretely, one study got 2 points; three studies got 3; twelve studies got 4; eleven studies got 5; seven studies got 6; three studies got 7 and five studies got 8 points. Five studies were not published because they were Master’s or Ph.D thesis. Twenty-seven studies mentioned control of temperature, including control of the room or water temperature. Nineteen studies adopted blinded building of model and seventeen mentioned assessment whether building model was successful. Random allocation to control group or acupuncture group and blinded assessment of outcome were described in 42 and 12 studies. No study reported inducing significant intrinsic neuroprotective activity because of anesthetic. Only one study described the sample size calculation. Fourteen studies reported statement of potential conflict of interests and eleven reported compliance with animal welfare regulations. The study quality and publication bias evaluation are shown in Table 2.

**Table 2:** Risk of bias of included studies

Study	(1)	(2)	(3)	(4)	(5)	(6)	(7)	(8)	(9)	(10)	Total
Bao 2014 [18]	√	√	√		√	√	√				6
Zeng 2008 [19]	√	√	√				√				4
Chen 2015 [20]	√	√	√		√	√	√	√	√		8
Chen 2006 [21]		√	√		√		√			√	5
Dai 2015 [22]	√	√	√	√		√					5
Hou 2013 [23]	√	√	√		√				√		5
Gao 2012 [24]		√	√		√					√	4
Huang 2010 [25]	√		√		√		√				4
Ji 2011 [26]	√	√	√				√				4
Jia 2011 [27]			√		√		√			√	4
Wang 2012 [28]	√	√	√	√	√		√				6
Lin 2008 [29]	√		√		√		√				4
Luo 2007 [30]	√		√								2
Ma 2009 [31]	√		√	√	√		√				5
Zhang 2014 [32]	√		√	√	√		√				5
Niu 2009 [33]	√	√	√				√				4
Su 2013 [34]	√		√				√				3
Tan 2014 [35]	√	√	√	√	√	√	√				7
Tang 2014 [36]	√		√				√				3
Wang 2013 [37]		√	√				√			√	4
Wang 2009 [38]			√		√		√			√	4
Hong 2014 [39]	√	√	√	√	√		√				6
Xu 2006 [40]	√		√	√			√				4
Xu 2007 [41]	√	√	√			√	√				5
Yi 2014 [42]	√		√	√	√	√	√				6
Yu 2014 [43]	√	√	√			√	√				5
Feng 2013 [44]	√	√	√	√			√				5
Li 2013 [45]	√		√	√			√				4
Wang 2013 [46]	√	√	√	√		√	√				6
Zheng 2009 [47]	√		√				√				3
Li 2012 [48]	√	√	√	√		√	√		√	√	8
Li 2014 [49]	√	√	√	√			√		√	√	7
Lee 2014 [50]	√	√	√	√		√	√		√	√	8
Zhu 2013 [51]	√	√	√	√			√			√	6
Lu 2014 [52]	√	√	√		√	√	√		√	√	8
Li 2012 [53]	√		√		√		√		√		5
Guo 2015 [54]	√	√	√				√		√	√	6
Jiang 2015 [55]	√	√	√	√			√		√	√	7
Shao 2008 [56]	√	√	√	√						√	5
Liu 2013 [57]	√	√	√				√		√		5
Li 2015 [58]	√	√	√	√		√	√		√	√	8
Lu 2008 [59]	√		√	√			√				4

(1) publication in a peer-reviewed journal; (2) statements describing control of temperature; (3) random allocation to treatment or control; (4) blinded building of model; (5) assessment whether building model is successful; (6) blinded assessment of outcome;(7) use of anesthetic without significant intrinsic neuroprotective activity; (8) sample size calculation; (9) compliance with animal welfare regulations; (10) declared any potential conflict of interest

### Effectiveness

**Fig. 2:**
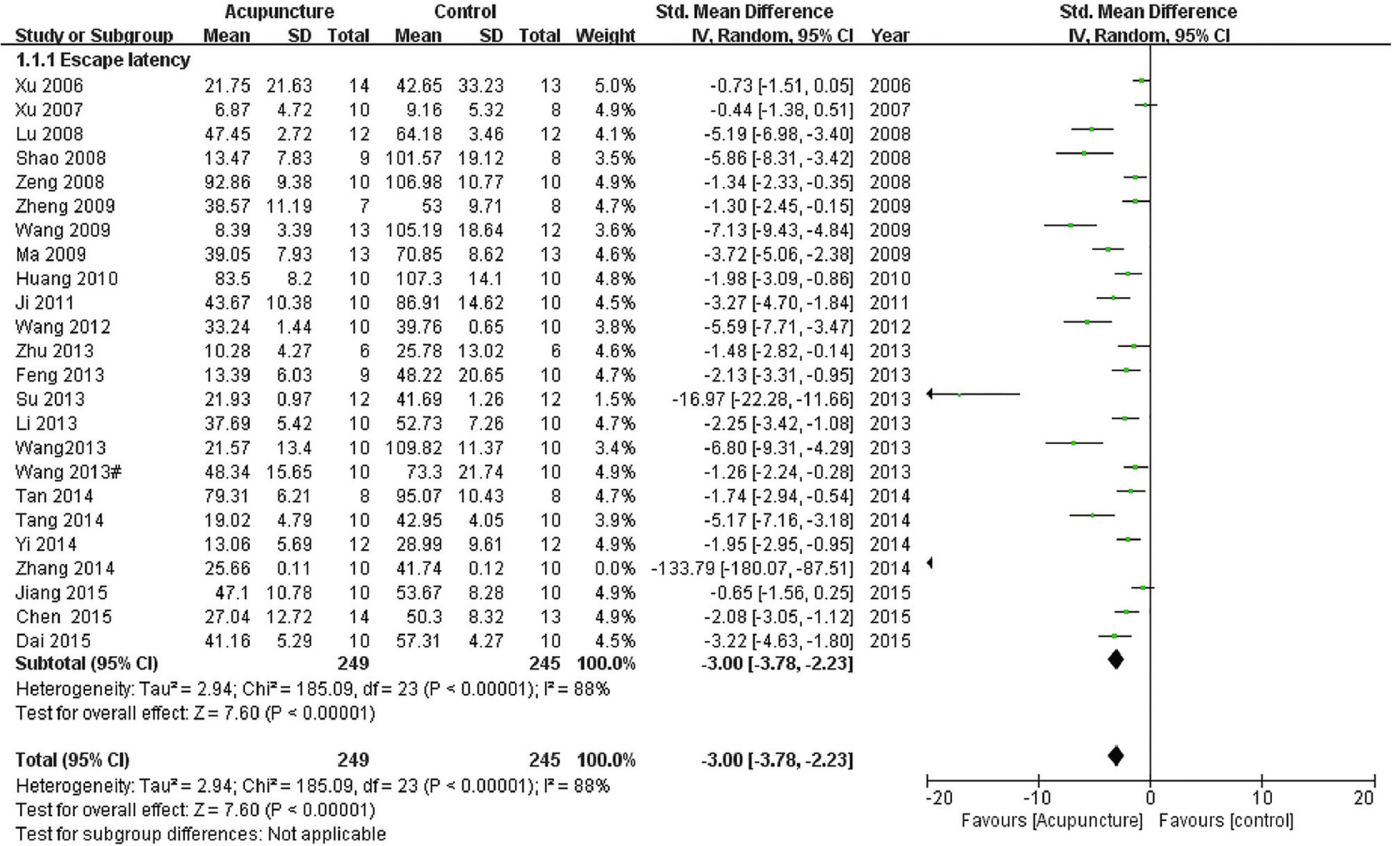
Pooled estimate of decreasing escape latency with acupuncture

Forty-one studies reported the impact of acupuncture on decreasing escape latency compared with the control group (*p* < 0.05 or *p* < 0.01). Twenty-four of them provided detailed data to show significant effectiveness of acupuncture compared with the control group (*n* = 494, SMD −3.00, 95 % CI: −3.78 ~ −2.23, *P* < 0.00001; heterogeneity ***χ***^2^ = 185.09, I^2^ = 88 %, Fig. 2). The remaining seventeen studies did not provide detailed data and just showed the data demonstrated in a graphical form.

**Fig. 3:**
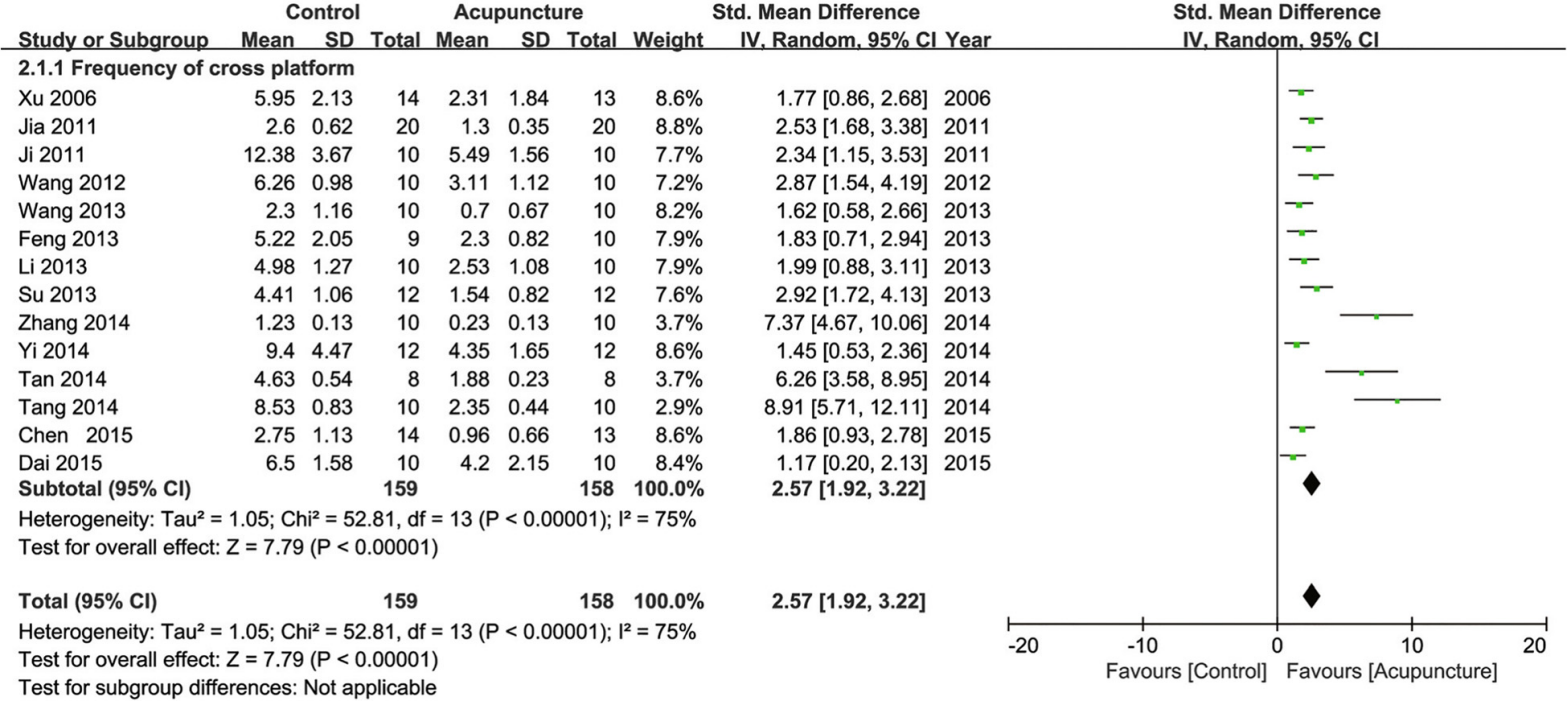
Pooled estimate of increasing frequency of cross platform with acupuncture

Eighteen studies reported the impact of acupuncture on increasing frequency of cross platform compared with the control group (*p* < 0.05 or *p* < 0.01). Fourteen of them provided detailed data to show significant effectiveness of acupuncture compared with the control group (*n* = 317, SMD 2.57, 95 % CI: 1.92 ~ 3.22, *P* < 0.00001; heterogeneity ***χ***^2^ = 52.81, I^2^ = 75 %, Fig. 3). The remaining four studies just showed the data demonstrated in the graphical form and failed for meta-analysis.

**Fig. 4:**
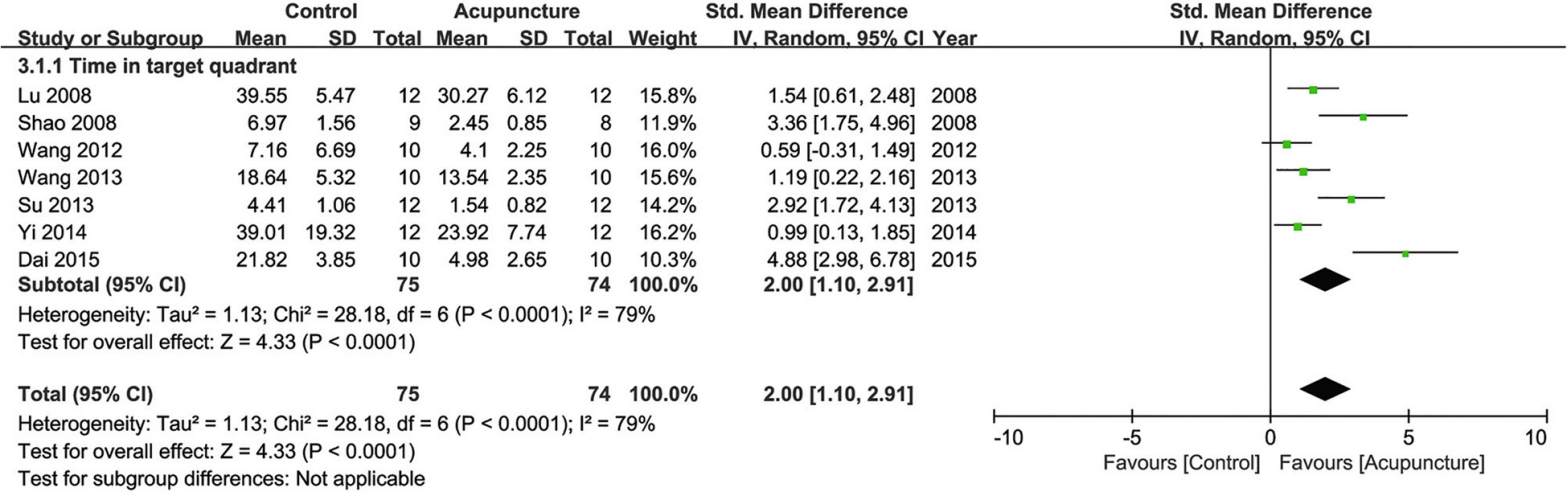
Pooled estimate of increasing time in target quadrant with acupuncture

Eleven studies reported the impact of acupuncture on increasing time in target quadrant compared with the control group (*p* < 0.05 or *p* < 0.01). Seven of them provided detailed data to show significant effectiveness of acupuncture compared with the control group (*n* = 149, SMD 2.00, 95 % CI: 1.10 ~ 2.91, *P* < 0.00001; heterogeneity ***χ***^2^ = 28.18, I^2^ = 79 %, Fig. 4). The remaining five studies just showed the data demonstrated in the graphical form.

### Signaling pathways

Several different signaling pathways were investigated to gain a better understanding of the underlying mechanism of acupuncture in the amelioration of learning and memory impairment. 39 of 42 included studies got detailed descriptions about possible mechanisms. It can be found that reduction nerve apoptosis and necrosis and suppression of oxidative stress are the main signaling pathways. A summary of proposed mechanism is shown in Table 3.

**Table 3:** Summary of proposed mechanisms

Study	Findings or proposed mechanisms
Bao 2014 [18]	• Reduced apoptosis of hippocampal neurons • Promoted restoration of damaged nerve cells
Chen 2015 [20]	• Reduced Nogo-A and NgR
Chen 2006 [21]	• Increased GABA • Reduced Glu
Dai 2015 [22]	• Increased NEP
Hou 2013 [23]	• Increased MR • Reduced GR
Gao 2012 [24]	• Increased DA, 5-HT and NE
Huang 2010 [25]	• Increased BDNF
Ji 2011 [26]	• Reduced MDA • Increased SOD
Jia 2011 [27]	• Increased Syp, PKC, NMDAR and PKC mRNA • Reduced mGluRs
Wang 2012 [28]	• Increased NOS • Reduced MAO
Lin 2008 [29]	• Increased NMDAR-2BmRNA
Luo 2007 [30]	• Decreased NO
Ma 2009 [31]	• Increased CTGF protein and mRNA
Zhang 2014 [32]	• Increased InsR mRNA
Niu 2009 [33]	• Increased SS and AVP
Su 2013 [34]	• Reduced MDA, P53 and P21 • Increased SOD
Tan 2014 [35]	• Increased GAP-43 and c-fos
Tang 2014 [36]	• Increased CHAT protein
Wang 2013 [37]	• Reduced GSK-3β
Wang 2009 [38]	• Reduced ET • Increased CGRP
Hong 2014 [39]	• Increased CX43, CX32 and CX36
Xu 2006 [40]	• Increased Bcl-2 protein • Reduced Bax protein and mRNA
Xu 2007 [41]	• Reduced IL-1β and TNF-α
Yi 2014 [42]	• Increased SOD, PKA and pCREB • Reduced MDA and c-fos
Feng 2013 [44]	• Increased GluR2
Li 2013 [45]	• Reduced MMP-2 and MMP-9
Zheng 2009 [47]	• Increased ChAT • Reduced TchE
Li 2012 [48]	• Reduced neuron loss
Li 2014 [49]	• Increased BDNF
Lee 2014 [50]	• Stimulated cholinergic enzyme activities • Regulated BDNF and CREB expression
Zhu 2013 [51]	• Up-regulated mTOR and eIF4E
Lu 2014 [52]	• Increased Fos expression
Li 2012 [53]	• increased Bcl-2mRNA • decreased caspase-3
Guo 2015 [54]	• Down-regulated Notch1 and Hes1 mRNA
Jiang 2015 [55]	• Increased the level of uptake rate of glucose
Shao 2008 [56]	• Regulated the amount of AVP, SS, and β-EP
Liu 2013 [57]	• Reduced MDA • Increased SOD
Li 2015 [58]	• Increased the pyramidal neuron number • Decreased the number of astrocytes
Lu 2008 [59]	• Increased NCAM and ST8SiaII/IVmRNA

*Nogo*-*A* neurite growth inhibitor-A, *NgR* neurite growth inhibitor receptor, *GABA* γ-aminobutyric acid, *Glu* glutamic acid, *NEP* neutral endopeptidase, *MR* mineralocorticoid receptor, *GR* glucocorticoid receptor, *DA* dopamine, *5*-*HT* 5-hydroxytryptamine, *NE* norepinephrine, *BDNF* brain-derived neurotrophic factor, *MDA* malondialdehyde, *SOD* superoxide dismutase, *SYP* synaptophysin, *PKC* Protein kinase C, *NMDAR* N-methyl-D-aspartate receptor, *mGluRs* metabolism glutanic acid acceptor, *NOS* nitric oxide synthase, *MAO* monoamine oxidase, *NO* nitric oxide, *CTGF* connective tissue growth factor, *InsR* insulin receptor, *SS* somatostatin, *AVP* arginine vasopressin, *GAP*-*43* Growth Associated Protein-43, *CHAT* choline acetyl transferase, *GSK*-*3β* glycogen synthase kinase-3β, *ET* endothelia, *CGRP* calcitonin gene-related peptide, *CX* connexin, *IL* interleukin, *TNF* tumor necrosis factor, *pCREB* phosphorylated cAMP-response element binding, *MMP* metal matrix proteinase, *ChAT* choline acetyltransferase, *TchE* total cholinesterase, *CREB* cAMP response element-binding protein, *mTOR* mammalian target of rapamycin, *eIF4E* eukaryotic translation initiation factor 4E, *NCAM* neural cell adhesion molecule

### Assessment of bias

**Fig. 5:**
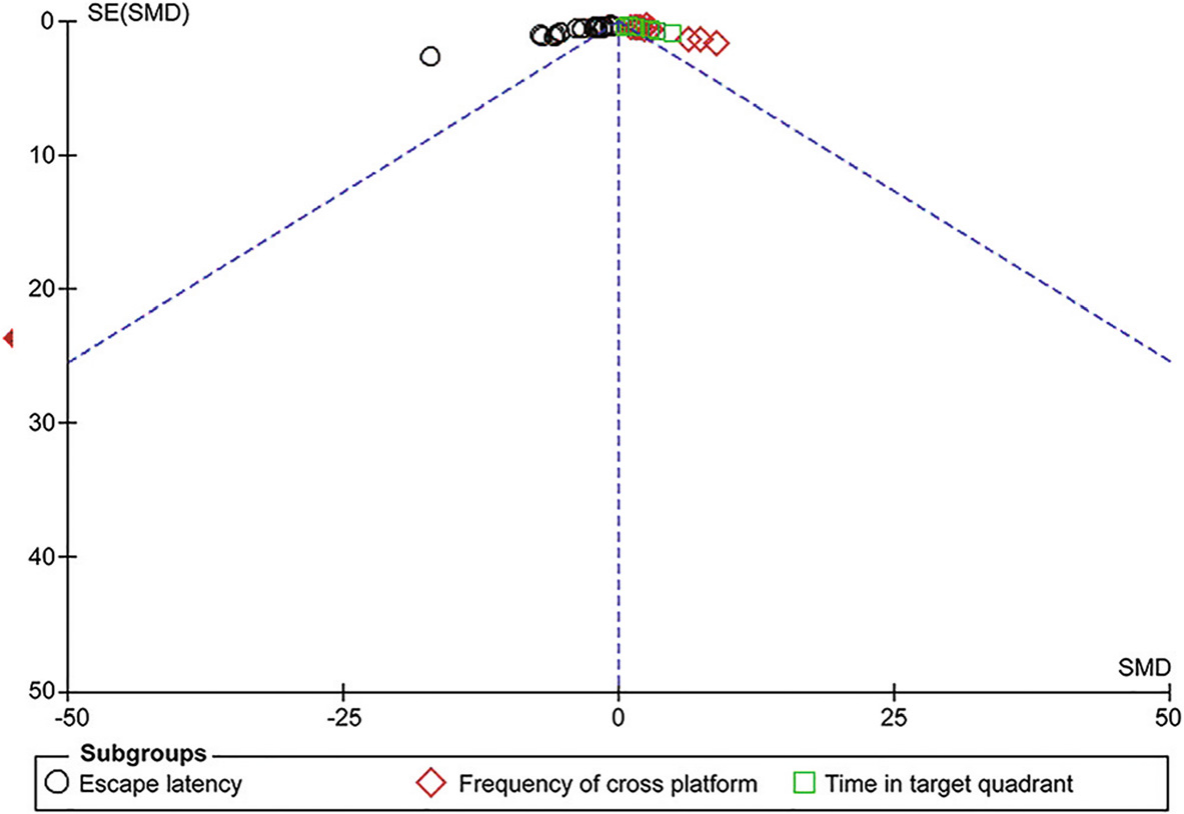
Funnel plot for effectiveness of acupuncture on escape latency, frequency of cross platform and time in target quadrant

The funnel plot was approximately symmetric for the effect of acupuncture on escape latency, frequency of cross platform and time in target quadrant (Fig. 5). Nevertheless, publication bias was still considered to be potential.

### Affecting factors of outcome indexes

**Fig. 6:**
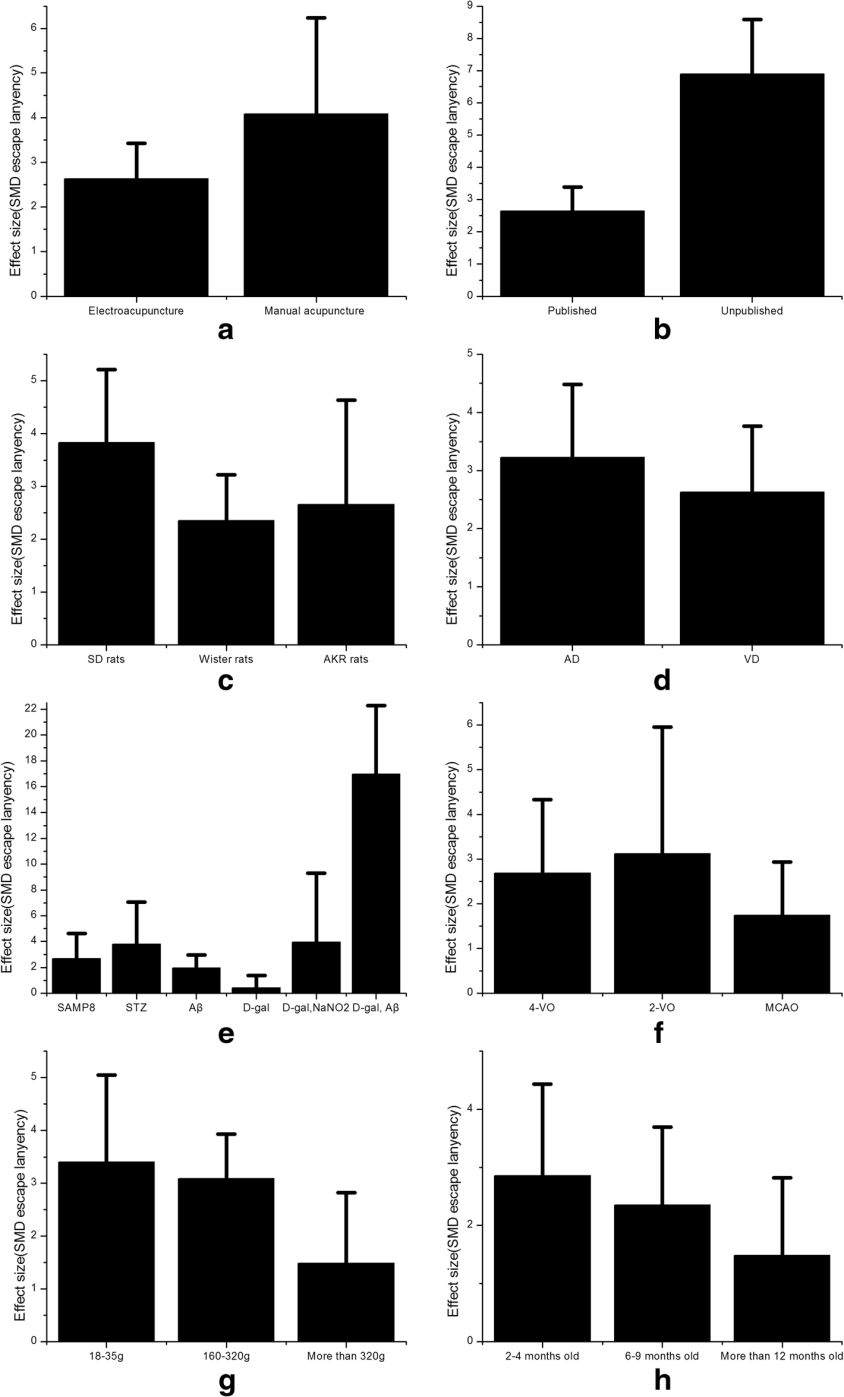
Subgroup analysis according to escape latency. **a** The effect of manual acupuncture and electroacupuncture on the estimate of improvement in escape latency. **b** The impact of published articles compared with unpublished articles on the estimate of improvement in escape latency. **c** The type of strain on the estimate of improvement in escape latency. **d** The sensitiveness of AD model compared with VD model on the estimate of improvement in escape latency. **e** The different ways making AD model on the estimate of improvement in escape latency. **f** The different ways making VD model on the estimate of improvement in escape latency. **g** The different weights on the estimate of improvement in escape latency. **h** The different age on the estimate of improvement in escape latency

In the subgroup analysis of escape latency, the efficacy of manual acupuncture was better than electroacupuncture (SMD −4.09, 95 % CI: −6.24 ~ −1.95, Fig. 6a). Compared with published articles, unpublished articles showed more obvious changes of escape latency caused by acupuncture treatment (SMD −6.89, 95 % CI: −8.68 ~ −5.29, Fig. 6b). According to different varieties of experimental animals, SD rats were more sensitive to acupuncture than Wister rats and AKR rats (SMD −3.83, 95 % CI: −5.21 ~ −2.45, Fig. 6c). AD and VD are the common diseases causing learning and memory impairment. After treatment, escape latency was further improved in AD animal model (SMD −3.23, 95 % CI: −4.48 ~ −1.99, Fig. 6d). There are many ways making AD and VD animal models. By analyzing different ways causing AD models, we found that AD models caused by D-gal and Aβ were more sensitive to acupuncture than other ways (SMD −16.97, 95 % CI: −22.28 ~ −11.66, Fig. 6e). By analyzing different ways causing VD models, we found that VD models caused by 2-VO were more sensitive to acupuncture than other ways (SMD −3.12, 95 % CI: −5.96 ~ −0.28, Fig. 6f). Different weight and age of rats were included in these studies. We found that rats whose weight ranges from 18 to 35 g (SMD −3.4, 95 % CI: −5.05 ~ −1.75, Fig. 6g) and rats whose age ranges from 2 to 4 months old (SMD −2.86, 95 % CI: −4.43 ~ −1.29, Fig. 6h) were more sensitive to acupuncture for improvement of frequency of escape latency.

**Fig. 7:**
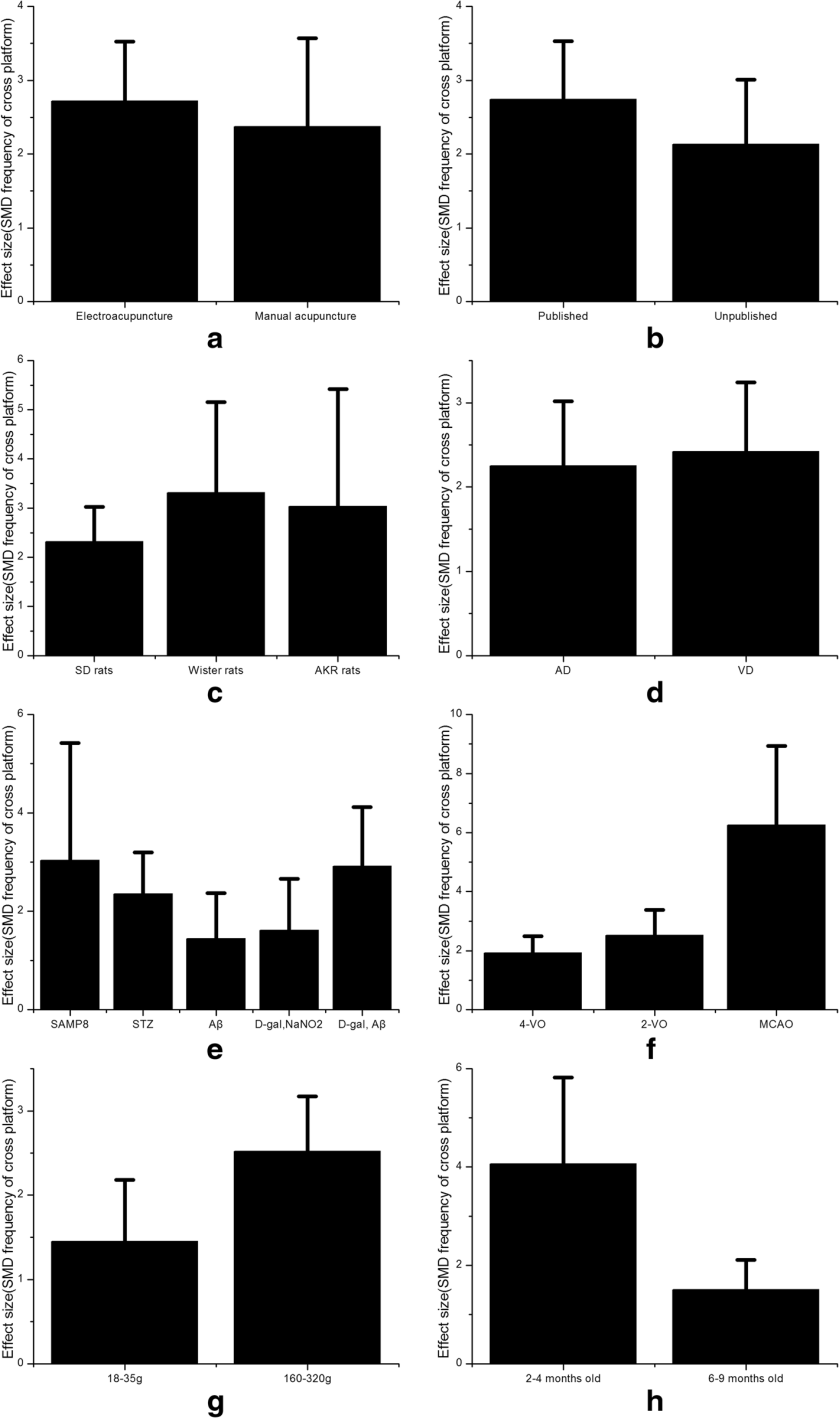
Subgroup analysis according to frequency of cross platform. **a** The effect of manual acupuncture and electroacupuncture on the estimate of improvement in frequency of cross platform. **b** The impact of published articles compared with unpublished articles on the estimate of improvement in frequency of cross platform. **c** The type of strain on the estimate of improvement in frequency of cross platform. **d** The sensitiveness of AD model compared with VD model on the estimate of improvement in frequency of cross platform. **e** The different ways making AD model on the estimate of improvement in frequency of cross platform. **f** The different ways making VD model on the estimate of improvement in frequency of cross platform. **g** The different weights on the estimate of improvement in frequency of cross platform. **h** The different age on the estimate of improvement in frequency of cross platform

In the subgroup analysis of frequency of cross platform, the electroacupuncture was more effective than manual acupuncture (SMD 2.72, 95 % CI: 1.91 ~ −3.52, Fig. 7a). Compared with unpublished articles, published articles showed more obvious changes of frequency of cross platform caused by acupuncture treatment (SMD 2.74, 95 % CI: 1.96 ~ 3.53, Fig. 7b). According to different varieties of experimental animals, Wister rats were more sensitive to acupuncture than SD rats and AKR rats (SMD3.31, 95 % CI: 1.47 ~ 5.16, Fig. 7c). Compared with AD models, VD models got more improvement of frequency of cross platform (SMD 2.42, 95 % CI: 1.60 ~ 3.24, Fig. 7d). By analyzing different ways causing AD models, we found that SAMP8 models were more sensitive to acupuncture (SMD 3.04, 95 % CI: 0.65 ~ 5.42, Fig. 7e). By analyzing different ways causing VD models, we found that MCAO models were more sensitive (SMD 6.26, 95 % CI: 3.58 ~ 8.95, Fig. 7f). Different weight and age of rats were included in these studies. We found that rats whose weight ranges from 160 to 320 g (SMD 2.52, 95 % CI: 1.86 ~ 3.17, Fig. 7g) and rats whose age ranges from 2 to 4 months (SMD4.06, 95 % CI: 2.30 ~ 5.83, Fig. 7h) were more sensitive to acupuncture for improvement of frequency of cross platform.

**Fig. 8:**
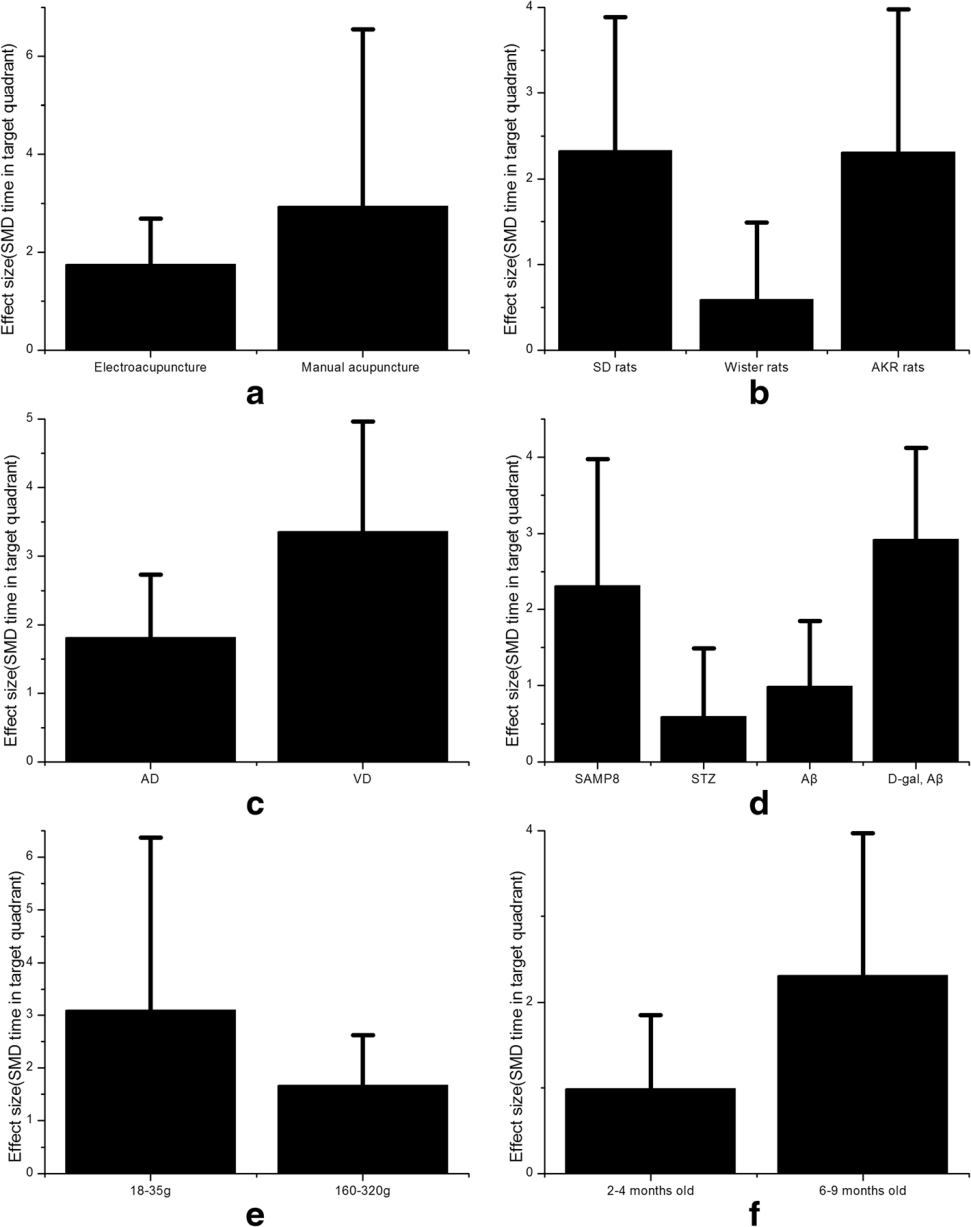
Subgroup analysis according to time in target quadrant. **a** The effect of manual acupuncture and electroacupuncture on the estimate of improvement in time in target quadrant. **b** The type of strain on the estimate of improvement in time in target quadrant. **c** The sensitiveness of AD model compared with VD model on the estimate of improvement in time in target quadrant. **d** The different ways making AD model on the estimate of improvement in time in target quadrant. **e** The different weights on the estimate of improvement in time in target quadrant. **f** The different age on the estimate of improvement in time in target quadrant

In the subgroup analysis of time in target quadrant, the efficacy of manual acupuncture was better (SMD 2.94, 95 % CI: −0.67 ~ 6.55, Fig. 8a). There were no unpublished articles using time in target quadrant as outcome indexes. According to different varieties of experimental animals, SD rats were more sensitive to acupuncture than Wister rats and AKR rats (SMD2.33, 95 % CI: 0.77 ~ 3.89, Fig. 8b). Compared with AD models, VD models got more improvement of time in target quadrant (SMD 3.36, 95 % CI: 1.75 ~ 4.96, Fig. 8c). By analyzing different ways making models of AD, we found that AD models caused by D-gal and Aβ were more sensitive to acupuncture (SMD 2.92, 95 % CI: 1.72 ~ 4.13, Fig. 8d). 4-VO was the only way to be included causing VD models and using time in target quadrant as one of outcome indexes. Different weight and age of rats were included in these studies. We found that rats whose weight ranges from 18 to 35 g (SMD 3.10, 95 % CI: −0.16 ~ 6.37, Fig. 7e) and rats whose age ranges from 6 to 9 months old (SMD 2.31, 95 % CI: 0.65 ~ 3.98, Fig. 7f) were more sensitive to acupuncture for improvement of time in target quadrant.

These results were mostly inconsistent in three subgroup analyses. It may be caused by studies of low quality, publication bias or other reasons.

## Discussion

To our knowledge, this is the first systematic review and meta-analysis to explore the efficacy of acupuncture for improving learning and memory in animal experiments with the results of Morris water maze test as the outcome measures. This systematic review and meta-analysis indicated that acupuncture could reduce time of escape latency, decrease frequency of cross platform and increase the time in target quadrant in animal model of learning and memory impairment. It suggests that acupuncture has a potential role in improving learning and memory impairment in animal models.

This review made a more detailed description of the acupuncture treatment procedure, including acupoint selection, stimulation methods, and treatment duration. The variation in the acupuncture protocol might contribute to the heterogeneity in treatment outcome between studies. The most common acupoints in acupuncture treatment for improving learning and memory were GV20 (baihui), ST36 (zusanli), GV14 (dazhui), BL23 (shenshu), BL17 (geshu) and CV17 (danzhong). GV20 and GV14 are Governor Vessel acupoints. BL23 and BL17 are Bladder Meridian of Foot-Taiyang acupoints. This result suggests the possible importance of Governor Vessel and Bladder Meridian of Foot-Taiyang for learning and memory. About one-third of included studies used manual acupuncture, and two-third used electroacupuncture. We have found that continuous waves, 2–4Hz of frequency and 1–2 mA of current density are the most commonly used stimulus parameters of electroacupuncture and twirling slowly is commonly used in manual acupuncture groups. Thus it can be seen that mild stimulation which means the reinforcing method in Traditional Chinese Medicine theory is adopted widely for improving learning and memory.

In the acupuncture study on animal model, setting sham acupuncture as control is very important and difficult. The importance of sham acupuncture is that it can help to clear the effectiveness of acupuncture after excluding placebo effects. While popular sham ways are mainly skin penetration. Except for placebo effects, it can also produce biological effects. Therefore, the true effect of acupuncture may be underestimated when compared to sham acupuncture [60].

Acupuncture has been known as an effective therapy for learning and memory impairment appearing in Alzheimer’s disease, vascular dementia, and so on [5–8]. The mechanism of acupuncture on improving learning and memory ability remains unclear. It is recognized that learning and memory are associated with cerebral cortex and hippocampus closely [40]. Modern medical research suggests that acupuncture may reduce nerve apoptosis and necrosis to protect cortex and hippocampus neurons through different aspects, which include decreasing the level of cytokines in hippocampus [41], adjusting the neurotrophic factors and cholinergic system [42, 47], inhibiting the expression of matrix metalloproteinase(MMP) [45], and so on. Additionally, it has been suggested that acupuncture can increase the activity of superoxide dismutase (SOD) and decrease the level of malondialdehyde (MDA) in brain to improve the antioxidant capacity and reduce brain tissue damage caused by free radical [58].

There are some limitations to this review. Firstly, our search only included Chinese and English articles and excluded those articles published in other languages. At the same time, we only included manual acupuncture and electroacupuncture and excluded some special acupuncture therapies, such as scalp acupuncture, auricular acupuncture, abdominal acupuncture, and so on. It may cause selective bias. Secondly, the total number of studies and the total sample size were too small for reliable. We have performed comprehensive literature search (six databases) and conducted extensive searches through other sources. But no more studies were found. Thirdly, articles which reported negative results may not be popular to publish so that the effectiveness of published articles would be better than those unpublished. Therefore, the effectiveness of acupuncture for improving learning and memory may be overstated. Fourthly, the quality of included studies was very low, so that it had significant impacts on the outcomes of the meta-analysis.

Based on the above limitations, more other language articles and special acupuncture treatment means should be included in the future systematic. Meanwhile, control of temperature, random allocation to treatment or control, blinded building of model, assessment successful model building and blinded assessment of outcome should be pay attention to in the future animal or clinical studies. Not only positive results, but also negative should be reported in the future animal or clinical studies.

In addition, some implications are also brought out after analyzing affecting factors of outcome indexes. Manual acupuncture showed more effective to escape latency and time in target quadrant than electroacupuncture. And they had roughly the same impact on frequency of cross platform. But in the present study, only 13 out of 42 studies performed manual acupuncture and the rest performed electroacupuncture. Because electroacupuncture is easier to control, standard and objectively measure than manual acupuncture [12], so it is used widely in clinical and experimental researches. Therefore, it remains unknown which means of acupuncture is more effective and convenient for people’s learning and memory impairment. Other impact factors do not show any universal regularity through histogram analysis. It need be solved by higher quality studies, lower publication bias, and so on.

## Conclusion

In animal model, acupuncture has a potential role in improving learning and memory ability. But it is still ambiguous that which stimulating mode (manual acupuncture or electroacupuncture) is more effective. Low quality of studies and larvaceous publication bias may reduce persuasiveness of positive results and should be solved in the future.

## Abbreviations

4-VO: 4-vessel occlusion
6-OHDA: 6-OH-dopamine
AD: Alzheimer’s disease
CFS: Chronic fatigue syndrome
CI: Confidence interval
CMS: Chronic mild stimulation
CNKI: China National Knowledge Infrastructure
MCAO: Middle cerebral artery occlusion
MDA: Malondialdehyde
MMP: Matrix metalloproteinase
OVX: Ovariectomy
PD: Parkinson’s disease
PTSD: Post-traumatic stress disorder
SD: Sprague–Dawley
SMD: Standard mean difference
SOD: Superoxide dismutase
SPS: Single prolonged stress
STZ: Streptozotocin
VD: Vascular dementia
VIP: Chinese Science and Technology Periodical Database
VPA: Sodium valproate
WD: Wilson disease
